# Case Report: Activated B-cell-diffuse large B-cell lymphoma

**DOI:** 10.12688/f1000research.134946.2

**Published:** 2024-07-08

**Authors:** Padmashri Kalmegh, Alka Hande, Madhuri Gawande, Swati Patil, Archana Sonone, Aayushi Pakhale

**Affiliations:** 1 Department of Oral and Maxillofacial Pathology and Microbiology, Sharad Pawar Dental College and Hospital, Datta Meghe Institute of Higher Education and Research, Sawangi (Meghe), Wardha, Maharashtra, 442004, India

**Keywords:** diffuse large B cell lymphoma, immunohistochemistry, lymphocytes, non-Hodgkin's lymphoma, oral cavity

## Abstract

Lymphomas of the oral and oropharyngeal regions are rather uncommon, and diagnosis can be challenging and confusing due to the multiple histological subgroups. Lymphomas are the third most common type of tumor in the head and neck region and are brought on by the lymphoreticular system. The two forms of lymphoma are Hodgkin’s lymphoma and non-Hodgkin’s lymphoma (NHL). Herein, we present a case report of oropharyngeal lymphoma. The female patient reported with a complaint of swelling over the palatal region for two to three months. An ulceroproliferative lesion was evident over the posterior palatal region. We diagnosed reactive lymphadenitis based on an incisional biopsy. To confirm the diagnosis and rule out other conditions, a punch biopsy followed by immunohistochemical studies were done. Features suggestive of activated B-cell-diffuse large B-cell lymphoma were confirmed. Among malignant lymphomas, diffuse large B-cell lymphoma is the most prevalent variety. Much progress has been made in recent years in understanding the molecular pathophysiology of this disease. In this case report, we aim to correlate the clinical presentation, histology features and immunohistochemical significance in order to promote early discovery, diagnosis, and treatment for a better prognosis of the patient.

## Introduction

Samuel Wilks coined the term “Hodgkin’s disease,” ^1^ Lymphomas are a diverse group of lymphoid malignancies with a range of clinical behaviour patterns and therapeutic responses. The histologic type, clinical variables, and more recently, molecular traits all affect the prognosis. ^2^ In terms of prevalence, lymphomas rank ninth among males and tenth among women worldwide. Lymphomas are the second most prevalent type of cancer in the head and neck region, and they rank third after squamous cell carcinoma and salivary gland cancers in the oral cavity. Even though lymphomas account for 3% to 5% of all reported cases in the head and neck region, they are the most prevalent non-epithelial malignant tumors in the same region. Oral cavity is an uncommon site for non-Hodgkin's lymphoma with the incidence rate of 0.1 to 5%. ^3^ Typically, no underlying cause of lymphoma is found in any specific occurrence. Nonetheless, other environmental, viral, and genetic risk factors for lymphoma have been discovered. Certain lymphomas have been linked to a number of infectious agents, including *Helicobacter pylori*, *Borrelia burgdorferi*, *Chlamydia psittaci*, and *Campylobacter jejuni.* ^4^ We adhere to the CARE reporting guidelines to report this Case report. ^5^

## Case report

**Figure 1.**
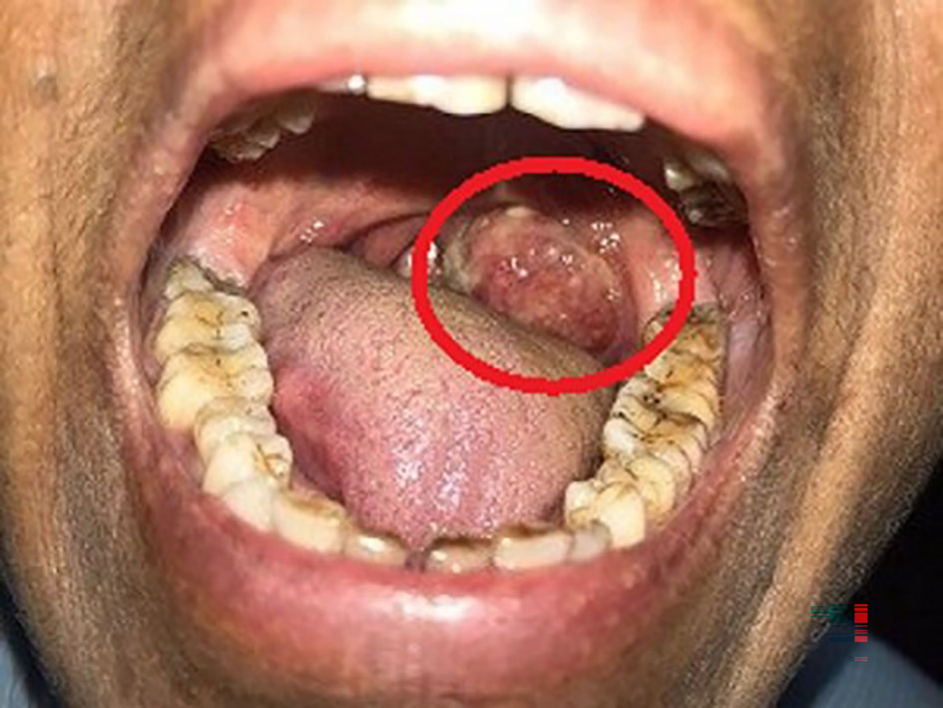
Clinical photo of the patient showing swelling on the posterolateral portion of the soft palate.

**Figure 2.**
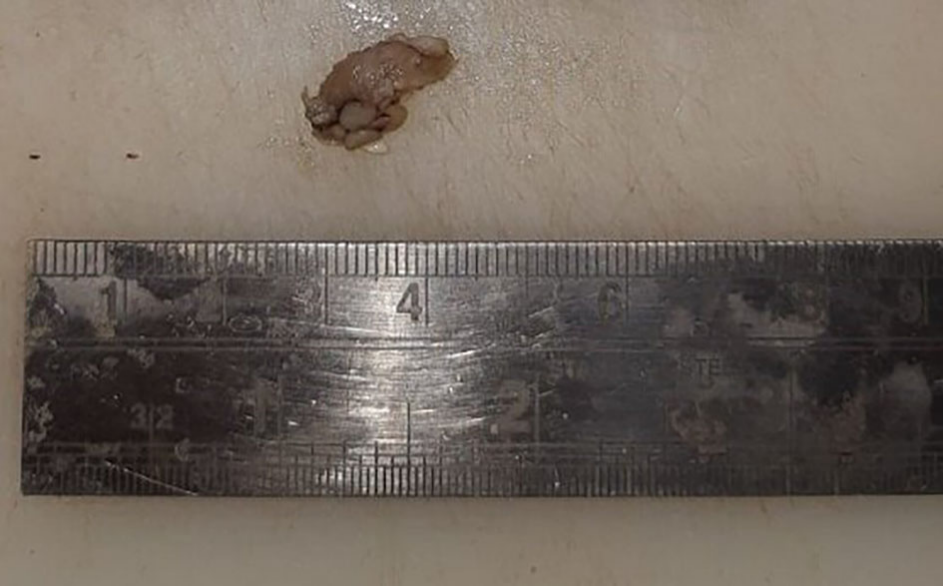
Punch biopsy specimen (2 × 1.7 cm).

A 63-year-old female, presented to the outdoor patient department of our institution in January 2023, with a complaint of a non-healing ulcer over the posterior palatal region for two to three months. She described no symptoms before two to three months but later observed swelling over the posterolateral region of the soft palate. Initially, it was 2 × 1 cm in size and increased to the current size of 4 × 3 cm approximately. She described difficulty in mastication and swallowing. A change in consistency and quantity of saliva was reported by the patient. No history of trauma was present. Intraorally, a single ulceroproliferative lesion present over the anterior faucial pillar was observed ( Figure 1, marked with a red circle). The lesion was oval, reddish pink in color, with ill-defined borders, soft to hard in consistency, nonmobile, and had an irregular surface. Uvula deviated to the left side. Lymph nodes in the left submandibular region were palpable, of size 1 × 2 cm, non-tender, and firm in texture. In December 2022, an incisional biopsy from the palatal mass was taken with a histopathological diagnosis of reactive lymphadenitis. This was followed by a punch biopsy from the same site ( Figure 2) in January 2023, with histopathological features suggestive of a lymphoproliferative lesion. No extraoral extension of the growth was evident.

### Radiographic features

**Figure 3.**
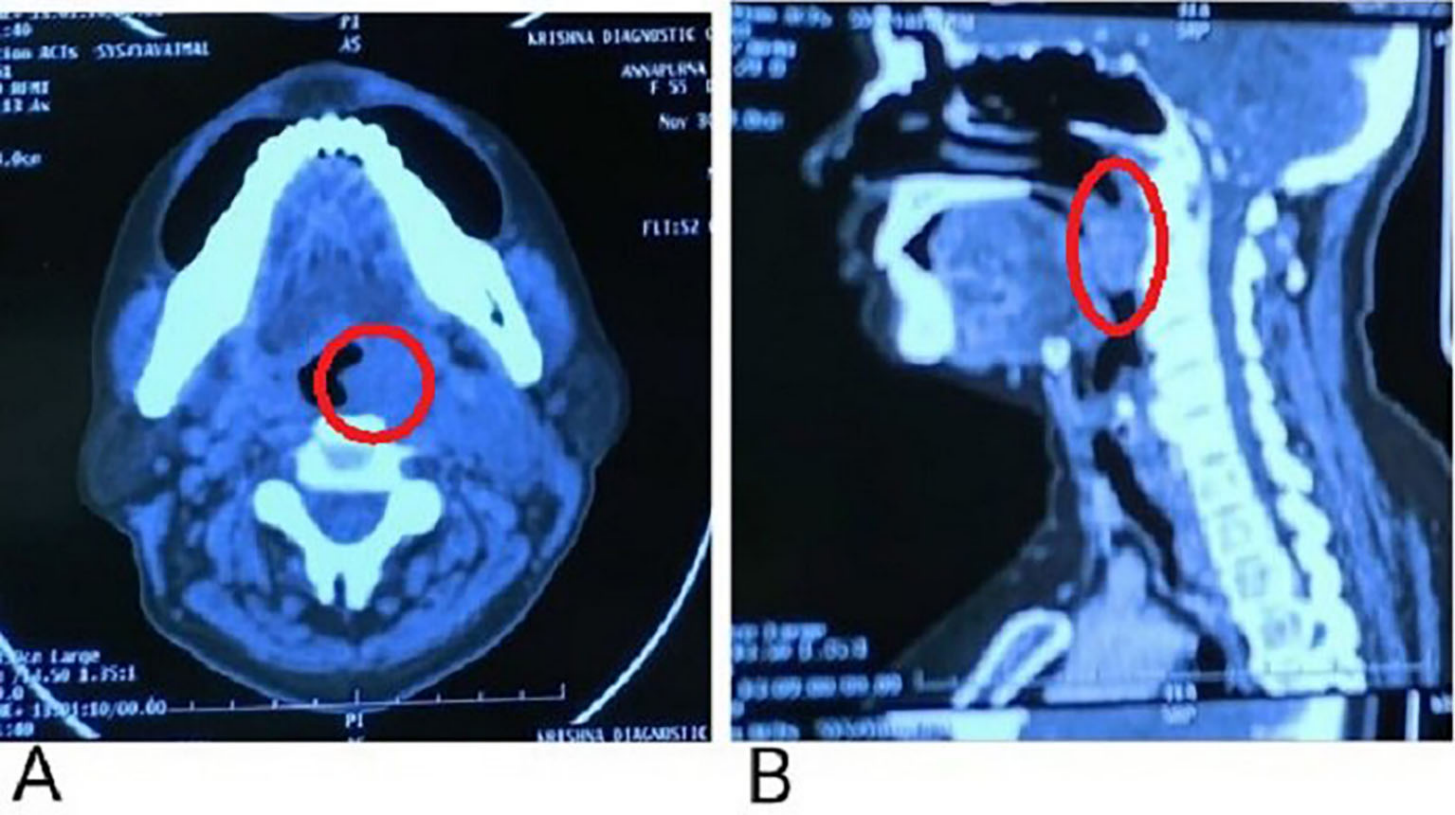
A: Non-enhancing necrotic areas noted in the left tonsillar fossa; B: Anteromedial extension abutting to the base of the tongue.

A contrast-enhanced computerized tomography (CECT) presented evidence of a soft tissue density lesion with few non-enhancing necrotic areas noted in the left tonsillar fossa measuring 2.2 × 2 × 2.7 cm ( Figure 3A, marked with a red circle) with the following extensions: a. Anteromedially extending into the oropharynx causing partial obstruction with non-visualization of uvula suggestive of involvement abutting the base of the tongue and vallecula ( Figure 3B, marked with a red circle). b. Posteroinferiorly involves the posterior pharyngeal wall. c. Laterally involving the peritonsillar space including the pharygobasillar fascia, styloglossus and stylopharyngeus, and the posterior belly of the digastric.

### Histological features

**Figure 4.**
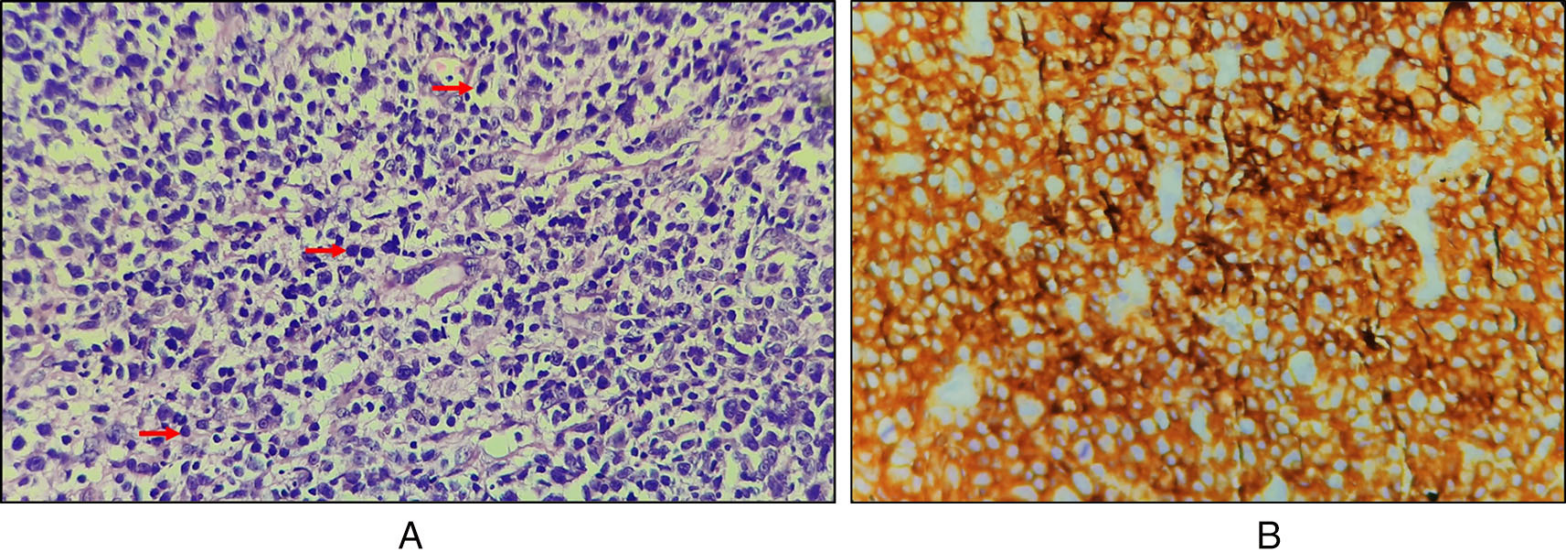
A: Diffuse infiltration of large lymphocytes (marked by red arrow); B: CD45 positive.

Haematoxylin and Eosin-stained sections showed predominantly a diffuse infiltrate of large-sized B cell population comprising of scant cytoplasm. The nucleus was double the size of the nucleus of a normal lymphocyte, vesicular chromatin, and prominent nucleoli ( Figure 4A, marked with red arrows). Complete effacement of normal lymphoid tissue architecture was present.

### Immunohistochemical findings

Immunohistochemical features with cytokeratin staining were negative, CD45 was strongly positive with an intensity score of 3, proportionate scores of 4 ( Figure 4B), and CD20 positive. On the basis of histopathological and immunohistochemical features, the lesion was suggestive of an activated B-cell (ABC) subset of diffuse large B-cell lymphoma (DLBCL).

### Treatment

In the present case, chemotherapy (cyclophosphamide, hydroxy doxorubicin, oncovin, and prednisone) was used to treat the patient. The patient continued receiving CHOP chemotherapy, and her overall health is improving. A four-month follow-up showed regression of the swelling. The patient is still on treatment.

## Discussion

DLBCL becomes less fatal and may even be cured with combination chemotherapy, which only successfully treats less than 50% of patients. The type of therapy will either be curative, where survival is the goal, or palliative, where the goal is to improve the patient’s quality of life, depending on the patient’s status. ^4^ Rituximab, cyclophosphamide, doxorubicin, vincristine, and prednisone (R-CHOP) is the standard treatment for DLBCL patients. Approximately 60–70% of DLBCL patients who continue this regimen are free of illness. ^6^

Malignant lymphomas are a category of neoplasms with variable degrees of malignancy that are derived from the lymphoid tissue’s fundamental cells, the lymphocyte, and histiocytes at any stage of development. ^7^ The classification of lymphomas originating from these normal lymphoid populations is challenging since the physiologic immunological roles of lymphocytes vary depending on lineage and degree of development. NHL and Hodgkin lymphoma (HL) are the two main categories into which lymphomas are frequently categorized. NHLs are a variety of lymphoproliferative malignancies that have a far higher propensity to disseminate to extranodal locations than Hodgkin’s lymphoma. ^2^ NHL, a class of neoplasms, occurs due to the uncontrolled proliferation of B-, T-, or Natural Killer (NK) cells. T-cell or NK cell-derived illnesses account for 10 to 15 percent of all NHL cases. ^8^ DLBCL, which accounts for 30–40% of cases in various parts of the world, is the most common NHL. It is most commonly seen in the sixth to seventh decade of life. ^9^ DLBCL is a diverse entity in terms of clinical presentation, genetic advances, therapeutic options, and prognosis. There was a significant advancement when gene expression profiling (GEP) was applied to further detect this variety and provide a rationale for categorizing patients in the study of DLBCL. DLBCL patients are divided into subtypes named germinal center B-cell-like (GCB), ABC, and 10–15% remain unclassified. The ABC subtype is derived from B cells that are undergoing plasma cell development. Patients with the ABC subtype often have the worst prognosis than those with the GCB subtype. ^10^ However, there is morphological and clinical heterogeneity in DLBCL. However, patients with GCB DLBCL still do considerably better than those with ABC DLBCL. ^11^ Expression of CD20 (cluster of differentiation antigens) in lymphoma cells is essential for both making an accurate diagnosis and creating a strategy for biological therapeutic therapy. CD20 antigen is expressed on the surface of neoplastic cells in the majority of B-cell lymphomas, albeit the degree of CD20 expression varies depending on the type of lymphoma and the degree of lymphoma B-cell differentiation. Hence, it is assumed that CD20 expression is high in DLBCL and hairy cell leukemia cells, and low in B-cell chronic lymphocytic leukemia (CLL). ^12^ Particularly, ABC DLBCLs express X-box binding protein-1 (XBP-1), a significant modulator of the secretory phenotype of plasma cells. ^13^ About 25% of ABC DLBCL samples taken from patients have BLIMP1-inactivating mutations in the PRDM1 gene. ^14^ By inhibiting the expression of the majority of mature B-cell differentiation genes, BLIMP1 promotes plasmacytic differentiation. ^15^ Consistent activation of the nuclear factor-kappa B (NF-κB) signaling pathway, which stimulates cell survival and proliferation while inhibiting apoptosis, is another pathogenic characteristic of ABC DLBCLs. ^16^ In order to ensure the most effective use of investigational treatments or currently accessible, frequently expensive therapeutic agents, pathologists must also evaluate the markers required to support their usage. Additionally, pathologists must prioritize sufficient and viable tumor tissue for high-throughput molecular analysis. ^17^^–^^21^

According to the location and histologic type, clinical presentation differs substantially. Biopsy in conjunction with immunological investigations of biopsy tissue is the only accurate way to identify and classify these lesions. ^22^ No single antibody has been useful for subdividing DLBCL or determining prognosis, according to research by Meyer *et al*. Because of this, many antibody combinations or methods have been created. ^23^ Histologically, the differential diagnosis of DLBCL includes Plasmablastic lymphoma (PBL), Burkitt lymphoma (BL), Oral mantle cell lymphoma (OMCL), Angioimmunoblastic T-cell lymphoma (ATL), Hodgkin lymphoma.

In PBL, the majority of patients pass away in less than a year due to its progressive clinical course. Histologically, PBL is made up of big malignant lymphoid cells with immunoblasts or plasmablasts and an immunophenotype specific to plasma cells. BL is made up of diffuse monomorphic growth of medium-sized cells with spherical nuclei with coarse chromatin, two or more distinct basophilic nucleoli, a ring of basophilic cytoplasm, and a high proliferation fraction. OMCL is a rare B-cell neoplasm that usually occurs in elderly males with preferential involvement of the palate and poor prognosis. ATL composed of diffuse polymorphic infiltration of small to medium sized lymphocytes. The tumor cells were reactive for T-cell markers (CD2, CD3, CD4, CD10). Similarly, lymphoblastic lymphoma is exceptionally rare and is predominantly of T-cell lineage. In HL, there is large atypical mononuclear, binucleared or multinucleated Reed-Sternberg/Hodgkin cells scattered within a background milieu of inflammatory cells (lymphocytes, eosinophils, histiocytes and plasma cells). Therefore, immunohistochemistry is pivotal for a definitive diagnosis. ^24^ IHC has enabled a better knowledge of the aetiology of lymphomas, and its careful use aids in the identification and characterization of immunophenotypes in the majority of lymphomas. The panel of markers is chosen based on morphologic differential diagnosis (no single marker is specific) and includes leukocyte common antigen (LCA), B-cell markers (CD20 and CD79a), T-cell markers (CD3 and CD5), and other markers such as CD23, bcl-2, CD10, cyclinD1, CD15, CD30, ALK-1, and CD138 (based on cytoarchitectural pattern). ^25^

For the differential diagnosis of 40 cases of oral NHL, Van der Waal *et al*. used the following antibodies: L26 (CD20, a pan B-cell marker), CD 79a (the immunoglobulin anchoring molecule, so a B-cell marker), CD3, and UCHL 1 (CD45RO), both pan T-cell markers, BerH2 (CD30), and Mib1 (staining primarily B cells). ^26^ Forty cases of oral cavity NHL were studied by Kemp *et al*. for sex, age, location, clinical presentation, and WHO histological subtype. They found that 98% of the lymphomas in their investigation had a B cell lineage, and the majority of these were histologically subtyped as diffuse large B cell lymphomas (58%). Our study is in accordance with Kemp *et al*., who used an immunohistochemical panel that included CD3, CD5, CD20, CD45RO, CD79a, leukocyte common antigen, Bcl-2, CD10 and CD23 to validate the lineage and help characterize the subtype. According to them, molecular testing is typically useful in determining genetic infiltration and seems to be a useful supplement to diagnosis. ^27^ In nonimmunocompromised patients, diffuse large B-cell lymphoma in the head and neck region was the most prevalent form observed, according to Hicks and Flaitz, 2001. ^28^ Hashimoto and Kurihara (1982), reviewed pathological characteristics of oral NHL and according to his nine cases and review of the literature, they concluded that B-cell lymphoma is the most common histotype in oral NHL. ^29^ Therefore, in order to diagnose a rare condition, this case report emphasizes the value of early biopsies of oral lesions and the use of a definitive marker among other available immunohistochemical markers.

### Limitations

The study has limitations due to the patient’s ongoing treatment and the relatively short follow-up time.

## Conclusions

Oral NHL is a very uncommon disorder that may cause localized swelling, inflammation, or discomfort in the vestibule, gingiva, or posterior hard palate. DLBCL is a genetically heterogeneous disease with equally different clinical manifestations. Hence, one can determine the malignant nature and prognosis of various kinds of lymphomas by correlating the histological characteristics of these entities. Pathologists should evaluate immunophenotypic and genetic markers that are useful for determining prognosis. In order for the patient to receive therapy at an early stage and have a favorable prognosis, a thorough evaluation of the patient and appropriate investigations are needed for a precise diagnosis.

## Consent

Written informed consent for publication of their clinical details and clinical images was obtained from the patient.

## Data Availability

All data underlying the results are available as part of the article and no additional source data are required. Zenodo: CARE checklist for ‘Case Report: Activated B-cell-diffuse large B-cell lymphoma’, https://www.doi.org.10.5281/zenodo.7918599. ^5^ Data are available under the terms of the Creative Commons Attribution 4.0 International license (CC-BY 4.0)
