# Supplementary material for: Case Report: Activated B-cell-diffuse large B-cell lymphoma
Source: F1000Res. 2024 Aug 2;12:769. Originally published 2023 Jul 3. [Version 3] doi: 10.12688/f1000research.134946.3 (PMC11292186; doi:10.12688/f1000research.134946.3)
Supplement: Peer review history

# Reviewer report: Reviewer response for version 3

**DOI:** 10.5256/f1000research.169876.r310395

**Published:** 2024-08-07

**Authors:** Junn-Liang Chang

**Affiliations:** 1 Department of Pathology and Laboratory Medicine, Taoyuan Armed Forces General Hospital, Longtan District, Taoyuan City, Taiwan

The author has provided reasonable, scientific and logical responses to the reviewer's suggestions. I have no comments.

Are enough details provided of any physical examination and diagnostic tests, treatment given and outcomes?

No

Is the case presented with sufficient detail to be useful for other practitioners?

Yes

Is sufficient discussion included of the importance of the findings and their relevance to future understanding of disease processes, diagnosis or treatment?

Yes

Is the background of the case’s history and progression described in sufficient detail?

Partly

Reviewer Expertise:

Cancer, Pathology, Immunology

I confirm that I have read this submission and believe that I have an appropriate level of expertise to confirm that it is of an acceptable scientific standard.

# Reviewer report: Reviewer response for version 2

**DOI:** 10.5256/f1000research.168733.r308966

**Published:** 2024-08-01

**Authors:** Dr. Akshay Dhobley

**Affiliations:** 1 Department of Oral Pathology and Microbiology, Government Dental College and Hospital, Nagpur, Maharashtra, India

well documented case report of an extremely rare lesion. Intra Orally large mass which was detected early and followed up carefully. All differential diagnosis have been mentioned both clinical as well as histological. The Immunohistochemical profile has been well documented which provides leading paths to the final diagnosis. The follow up of patient post surgical, has been mentioned.

Are enough details provided of any physical examination and diagnostic tests, treatment given and outcomes?

Yes

Is the case presented with sufficient detail to be useful for other practitioners?

Yes

Is sufficient discussion included of the importance of the findings and their relevance to future understanding of disease processes, diagnosis or treatment?

Yes

Is the background of the case’s history and progression described in sufficient detail?

Yes

Reviewer Expertise:

Oral Diseases, Oral Cancer, Oral Lichen planus, Odontogenic cysts  & tumors

I confirm that I have read this submission and believe that I have an appropriate level of expertise to confirm that it is of an acceptable scientific standard.

# Reviewer report: Reviewer response for version 2

**DOI:** 10.5256/f1000research.168733.r308227

**Published:** 2024-07-31

**Authors:** Puneeta Vohra

**Affiliations:** 1 SGT University, Gurugram, India; 2 ORAL MEDICINE & RADIOLOGY, SGT UNIVERSITY, gurgaon, gurgaon, India

1. Understanding the diverse subtypes and their management is crucial for optimizing patient care. Author needs to explain the the rarity of there case 

 2. Diagnosis Diagnosis involves a combination of clinical evaluation, imaging, and pathological assessment.

 PET Scans: Essential for staging, assessing disease extent, and evaluating treatment response.(not included) 

 The article is well written and should be published. Incase there is PET scan of this case it should be included in the case report that will highlight about involvement of any other organ single multi organ involvement has been identified in many cases. If the PET scan has not been done it should be mentioned in discussion.

Are enough details provided of any physical examination and diagnostic tests, treatment given and outcomes?

Yes

Is the case presented with sufficient detail to be useful for other practitioners?

Yes

Is sufficient discussion included of the importance of the findings and their relevance to future understanding of disease processes, diagnosis or treatment?

Yes

Is the background of the case’s history and progression described in sufficient detail?

Yes

Reviewer Expertise:

oral medicine, oral oncology, oral radiology, oral infections, oral cysts and tumours, hiv/aids, mucocutaneous disorders

I confirm that I have read this submission and believe that I have an appropriate level of expertise to confirm that it is of an acceptable scientific standard.

# Response

**Published:** 2024-07-31

**Authors:** Padmashri Kalmegh

**Affiliations:** Oral Pathology & Microbiology, Sharad Pawar Dental College and Hospital,, Wardha, Maharashtra, India

Thank you for your comments

# Reviewer report: Reviewer response for version 1

**DOI:** 10.5256/f1000research.148043.r288731

**Published:** 2024-06-28

**Authors:** Soundarya Ravi

**Affiliations:** 1 Departments of Pathology, Jawaharlal Institute of Postgraduate Medical Education and Research, Pondicherry, India

This case report discusses about rare presentation of an extranodal  DLBCL in the oral cavity in an elderly female presenting with a palatal mass. There are many issues that needs to be addressed in this manuscript.

 1. Abstract: The age of the patient is not mentioned in the abstract. 

 2. In both the abstract and case report, the authors have failed to mention the site of biopsy. Was it the palatal mass or the left submandibular lymph nodes? If I assume that the biopsy was taken from the palatal mass, then why was it reported as reactive lymphadenitis? Whether any IHC was performed in the initial biopsy? Could you add images of the initial biopsy? Was the initial biopsy not representative of the lymphoma? Kindly clarify in detail.

 3. Introduction: The first line mentioning the history of Hodgkin lymphoma seems redundant in this context. Similarly, the last line also mentions adherence to CARE reporting guidelines, which I feel is unnecessary.

 4. Case report: It would be better if the radiological findings were explained before describing the histopathology. Being written from a pathologist's perspective, I feel the histopathological description, morphological differential diagnosis were not described elaborately when compared to the radiology.

 In the morphology. how can a pathologist say "atypical B cell population". It should be mentioned only after IHC findings.

 5. IHC: What IHC panel was performed for this patient? There was only mention of CD45 and CD20. On what basis, you were able to diagnose it as Activated B-cell subtype? Please clarify. Add corresponding IHC images also. 

 6.  Treatment: The first paragraph is from the literature and should be included in the description and not in the case report.

 7. Discussion: The discussion elaborates on the types of lymphoma, CD20 expression in other lymphomas and molecular subtypes of DLBCL, which could be cut down. It would be better if the authors could discuss about the morphological differential diagnosis of DLBCL in this site and how to exclude them. They can also include a paragraph of the role of IHC in making accurate diagnosis and IHC approach for oral cavity undifferentiated tumors. 

 8. Nowhere in the article, I could find the incidence of lymphoma in oral cavity and what are the various lymphomas that could present with oral cavity lesion.

 9. Histopathology images: High-power images are required with better clarity to appreciate the morphology. If there was a focus which shows lymphoma and overlying skin ulceration, it would be better representative.

Are enough details provided of any physical examination and diagnostic tests, treatment given and outcomes?

Partly

Is the case presented with sufficient detail to be useful for other practitioners?

Partly

Is sufficient discussion included of the importance of the findings and their relevance to future understanding of disease processes, diagnosis or treatment?

No

Is the background of the case’s history and progression described in sufficient detail?

Partly

Reviewer Expertise:

Hematopathology, Leukemia, Lymphoma, Myeloproliferative neoplasms, Flow cytometry

I confirm that I have read this submission and believe that I have an appropriate level of expertise to confirm that it is of an acceptable scientific standard, however I have significant reservations, as outlined above.

# Response

**Published:** 2024-07-01

**Authors:** Padmashri Kalmegh

**Affiliations:** Oral Pathology & Microbiology, Sharad Pawar Dental College and Hospital,, Wardha, Maharashtra, India

The corrections are as follows,

1. In the abstract age of the patient is added.
2. In the abstract and case report site of the biopsy has been mentioned.
3. The first line from the introduction is removed.
4. Radiological findings are updated before histopathological findings.
5. Removed the word ‘atypical’ from the histopathological findings.
6. Morphological differential diagnosis is updated in the discussion section.
7. 1 ^st^ paragraph from the treatment is added to the discussion.
8. Incidence of lymphoma in the oral cavity is added.
9. Histopathological and CD45 IHC images are replaced.
10. The role of the IHC marker in the diagnosis of NHL is added.

Clarifications

 Comment 2 – Although the site of the biopsy was from the palatal region, on microscopy it showed numerous atypical cells with pleomorphic features. Hence the diagnosis of reactive lymphadenitis was made. IHC was not performed for the initial biopsy. The initial biopsy was taken from OPD so we could not take photographs for the same.

 Comment 3 – The last line of the introduction is added as per the guidelines of the journal.

# Reviewer report: Reviewer response for version 1

**DOI:** 10.5256/f1000research.148043.r183654

**Published:** 2023-07-17

**Authors:** Junn-Liang Chang

**Affiliations:** 1 Department of Pathology and Laboratory Medicine, Taoyuan Armed Forces General Hospital, Longtan District, Taoyuan City, Taiwan

Reviewer’s comments to Author:

1. Is there any laboratory test data that can be presented in this case report? Such as EBV or Serology detection?
2. Does the patient have enlarged lymph nodes on other imaging?
3. Physical examination for lymph node lesions in other parts?
4. What should be included in the imaging differential diagnosis and clinical differential diagnosis? Can it be determined that he has primary lymphoma?
5. Is there detection of EBV-related staining or molecular detection in pathological diagnosis?
6. What are the learning objectives of this case report?

Are enough details provided of any physical examination and diagnostic tests, treatment given and outcomes?

No

Is the case presented with sufficient detail to be useful for other practitioners?

Yes

Is sufficient discussion included of the importance of the findings and their relevance to future understanding of disease processes, diagnosis or treatment?

Yes

Is the background of the case’s history and progression described in sufficient detail?

Partly

Reviewer Expertise:

Cancer, Pathology, Immunology

I confirm that I have read this submission and believe that I have an appropriate level of expertise to confirm that it is of an acceptable scientific standard, however I have significant reservations, as outlined above.

# Response

**Published:** 2023-11-20

**Authors:** Padmashri Kalmegh

**Affiliations:** Oral Pathology & Microbiology, Sharad Pawar Dental College and Hospital,, Wardha, Maharashtra, India

Respond to the reviewer's comment -

 1. No other laboratory test data is presented in the case report.

 2. Yes, a few sub-centimetric to centimetric enlarged lymph nodes in bilateral submandibular, jugulo-digastric regions are seen.

 3. Physical examination for lymph nodes at other sites including submental, submandibular, supraclavicular, and upper jugulodigastric regions was done.

 4. Clinical differential diagnosis may include- Squamous cell carcinoma, Round cell tumor, Reactive lymphadenitis, Pleomorphic adenoma etc.

 Radiographic differential diagnosis may include - Oropharyngeal squamous cell carcinoma

 5. No EBV-related staining was done. For molecular detection of EBV - immunohistochemistry CD45 and CD20 was done as mentioned before.

  6. Learning objectives includes - There are many histological subtypes of lymphoma; therefore to differentiate and specify this presented rare subtype, case report would be useful.

# Response

**Published:** 2024-07-01

**Authors:** Padmashri Kalmegh

**Affiliations:** Oral Pathology & Microbiology, Sharad Pawar Dental College and Hospital,, Wardha, Maharashtra, India

**Comment 1** – Other laboratory investigations that were performed include a complete hemogram, hormonal profile, and immunohistochemistry.

**Comment 2** -  Lymph nodes in the left submandibular region were palpable, of size 1 × 2 cm, non-tender, and firm in texture.

**Comment 3 & 4– **Radiological differential diagnosis may include benign hyperplasia **, **salivary gland tumor, lymphadenopathy, and nasopharyngeal carcinoma.

**Comment 5 - **there were no EBV-related alterations seen on pathological examinations

**Comment 6 – **The presented case is rare and in this case report we have mentioned its clinical features, aetiology, histopathological features, and various IHC  markers in detail that may be the addition to the knowledge of the reader.
